# Assessing dosimetric benefit from daily online adaptive radiation therapy for esophageal cancer

**DOI:** 10.1002/acm2.70244

**Published:** 2025-09-23

**Authors:** Antonia Kubiatowicz, Michael Sherer, Ayaka Owaga, Andrew Sharabi, Xenia Ray

**Affiliations:** ^1^ Department of Radiation Medicine University of California ‐ San Diego La Jolla California USA; ^2^ Department of Radiation Oncology and Image‐Applied Therapy Kyoto University Kyoto Japan

**Keywords:** esophagus, online adaptive

## Abstract

A cohort of 19 esophageal cancer patients treated at our institution were analyzed to assess the clinical feasibility and dosimetric benefit from daily adaptive radiation therapy (ART). An Ethos‐planning template was developed to generate the initial ethos reference plan and daily adaptive plans using 9‐field IMRT for reduced on‐treatment optimization time. The template included relevant OAR goals and used an institutionally validated esophagus RapidPlan model. Clinical planning margins were used. Ethos‐generated plans were validated against the clinically‐approved plans which used 2‐arc VMAT. Weekly on‐treatment CBCT images were used to simulate doses from daily nonadaptive and adaptive techniques using the Ethos 2.0 Emulator. Timing data was recorded for each fraction. Dose metrics from institutional guidelines were compared between standard‐of‐care (SOC) and adaptive plans using a Wilcoxon signed rank test; these included mean dose for heart, lungs and liver, lungs V20Gy, and max dose for heart, spinal canal, and stomach. All Ethos template‐generated plans were found equivalent to clinical plans. The daily adaptive workflow required 14.1 ± 6.8 min. Dosimetric improvements were variable by patient. Some patients experienced large metric reductions while others saw very minor benefits if any. For a sub cohort of patients that received high benefit, statistically significant (*p* < 0.05) reductions in the lung mean, lung V20, heart mean, and heart V30 and V40 were observed. From these results we conclude that Ethos ART is feasible in terms of plan quality and on‐treatment time for esophageal cancer. ART can produce significant normal tissue dose reductions; however, not all patients benefited equally. Thus, identifying high‐benefit patients prior to treatment is necessary. Preliminary modeling results suggest this can be done prospectively, but these models are internally validated and require larger datasets to fully develop.

## 1 INTRODUCTION

Esophageal cancer affects approximately 22 000 people per year and many patients receive external beam radiation therapy (RT) as part of their curative treatment in the United States.^1^ When delivering RT, the goal is to deliver a therapeutic dose to the tumor while minimizing dose to surrounding normal tissues. For esophageal treatments, these normal tissues typically consist of the heart, lungs, stomach, liver, and spinal cord. To achieve this, inverse optimization^2^ is used on delineated targets and OARs from a planning simulation computed tomography (CT) scan to create a radiation treatment plan. In standard of care (SOC) image guided radiation therapy (IGRT), this plan is then treated each day by registering the patient's planning CT to a daily image acquired using on‐board cone beam computed tomography (CBCT). However, sometimes patient anatomy can change over the course of treatment and the targets or OARs may have deformed or changed position.^3^, ^4^ These changes can risk targets being under covered or OARs receiving higher doses than planned which puts the patient at risk of locoregional recurrence as well as toxicity.^5^

Daily online adaptive radiotherapy (ART), initially proposed by Yan et al.^3^ is a unique approach which allows for the original plan to be re‐optimized on the daily anatomy from each fraction. Several commercial technologies exist for performing this additional step including the Varian Ethos, Elekta Unity MR‐Linac, and the ViewRay MRIdian.^6^

Despite its advantages, online ART is time‐intensive and requires additional personnel to contour the daily anatomy at each treatment procedure, review these contours, and perform pre‐treatment QA. This lengthens the overall appointment times which can be difficult for clinic management and can be challenging for patients who must remain still for the duration and may be in an uncomfortable position. Previous studies have looked at strategies to mitigate these issues^7^ however there is still an increased resource burden to perform these treatments. Therefore, it is imperative that patients receiving this treatment be those that will receive the highest benefits from it.

**FIGURE 1:**
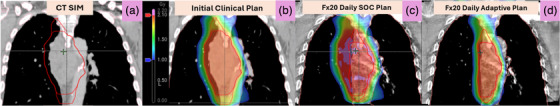
Example adaptive RT benefit for 1 subject. (a) Subject's initial CT simulation scan with the original physician‐drawn PTVs. (b) Initial clinically approved plan for 1 fraction (out of 25). (c) Dose distribution for the calculated standard of care plan at Fx20 showing extra dose to the lung. (d) Dose distribution for the adaptive plan at Fx20 showing improved conformality. Between Fx1 and Fx20 the tumor shrank dramatically, resulting in a large volume of prescription dose being unnecessarily delivered to the lung, if adaptation was not used.

Esophageal cancer patients have the potential to receive large benefits due to the response of the tumors over the course of radiotherapy (Figure 1). In the CROSS trial, tumor volume regression was shown to begin as early as after the first week, with a significant decrease in tumor volumes every subsequent week; there was a stepwise decrease from 100% to 91% at the second week, 81 % at the third, 77% at the fourth, and 72% at the fifth week.^8^ When these targets regress, studies have observed that OARs end up in the initial gross tumor volume (GTV).^9^ Furthermore, up to 10% of patients treated with CRT experience cardiac toxicity, and 7% experience grade 2 or higher pneumonitis following their treatments.^9^

Previous studies looking at the potential of offline ART to benefit these patients were performed, but all used 3D‐CRT beam arrangements.^10^ This is the first study, to our knowledge, to investigate the use of daily ART for esophageal patients on an existing commercially available ART system using updated IMRT planning techniques. The purpose of this study was to determine if ART for esophageal cancer patients is feasible and dosimetrically beneficial by simulating adaptive sessions for previously treated patients. A secondary goal was to evaluate if high‐benefit patients could be predicted based on information available in their initial CT simulation scans and contours.

## 2 METHODS

### 2.1 Patient selection

All data from this study was collected under approval of our institutional review board (IRB 200135). Data for 19 esophageal cancer patients who underwent treatment at our institution between August 2022 and August 2023 were collected. This data included the planning CT, clinically approved plan with RT structure set, and five on treatment cone beam CTs (CBCTs) taken at weekly intervals. Of these patients, two had stage 1 disease, two stage 2, eight had stage 3, and seven had stage 4. Eleven patients were treated to 50 Gy in 25 fx and eight were treated to 45 Gy in 25 fx with a simultaneous integrated boost to 50 Gy to the gross disease. The majority of patients’ disease was located in the lower third of the esophagus (16). One patient had disease in the upper third, and two in the middle third.

### 2.2 Ethos reference plan generation

To evaluate the online adaptive process, we used an Ethos emulator which allows both reference plan generation (aka initial treatment planning) and simulating online adapted sessions using previously acquired CBCT images. Ethos uses a unique plan optimization algorithm, called the intelligent optimization engine, for both the initial reference plan and the online plan. Clinical plans had been optimized in Eclipse V16.01 using the Photon Optimizer V16.01. algorithm. Although Ethos 2.0 can generate both VMAT and IMRT plans, institutional policy is to plan adaptive treatments with static‐angle IMRT to reduce the plan optimization time during the on‐treatment adaptive sessions. For reference, an IMRT plan calculates in approximately 3–5 min while a VMAT plan calculates in 10–15 min. The additional time on the couch for VMAT optimization has been deemed to extend the appointment to an unacceptable length of time by our institution when we can achieve our clinical goals with IMRT and thus all online adaptives are planned this way by our institution. This differed from the 2 arc institutional standard beam arrangement for esophageal cancer which was used for all the clinically treated plans in our patient cohort. Thus, the first step in our study was to evaluate our ability to create high‐quality plans on the patient's planning CT with the Ethos optimizer and static field IMRT, called reference plans. A planning directive template was developed using the Ethos version 2.0 emulator (Varian Medical Systems, Palo Alto, CA) prioritizing clinical goals. The planning directive template was based upon institutional standards and guidelines. Unfortunately, the manufacturer lost the original templates after the completion of this study so they cannot be included. Instead, we have included a table of our institutional guidelines for esophagus treatments as supplemental S1. Although the goals and templates of the planning directive were based on these constraints, our planning goals were slightly different to ensure the generation of clinically acceptable plans while allowing the robustness to the change of daily anatomy. In general, PTV coverage is prioritized unless exceeding a hard constraint on a critical OAR such as the spinal canal.

All Ethos reference plans were compared against their corresponding clinically approved plans to ensure they met or exceeded the plan quality achieved by the clinical plan prior to simulating adaptive treatments. This was assessed through evaluation of the following metrics: PTV hotspot, stomach V45, spinal canal D0.03cc, lung mean, lung V20, lung V5, heart V5, V20, V30, V40, and V50, Liver V30, and liver mean dose. These metrics were taken from Liu et al.^5^ PTV coverage was normalized to V100% = 95% for all plans both clinical and Ethos generated as per our institutional standard and to maintain fair comparison. After validation, these Ethos references plans were used to determine the daily dose from a standard of care (SOC) nonadaptive treatment approach versus the daily adaptive approach.

### 2.3 Adaptive simulation

**FIGURE 2:**
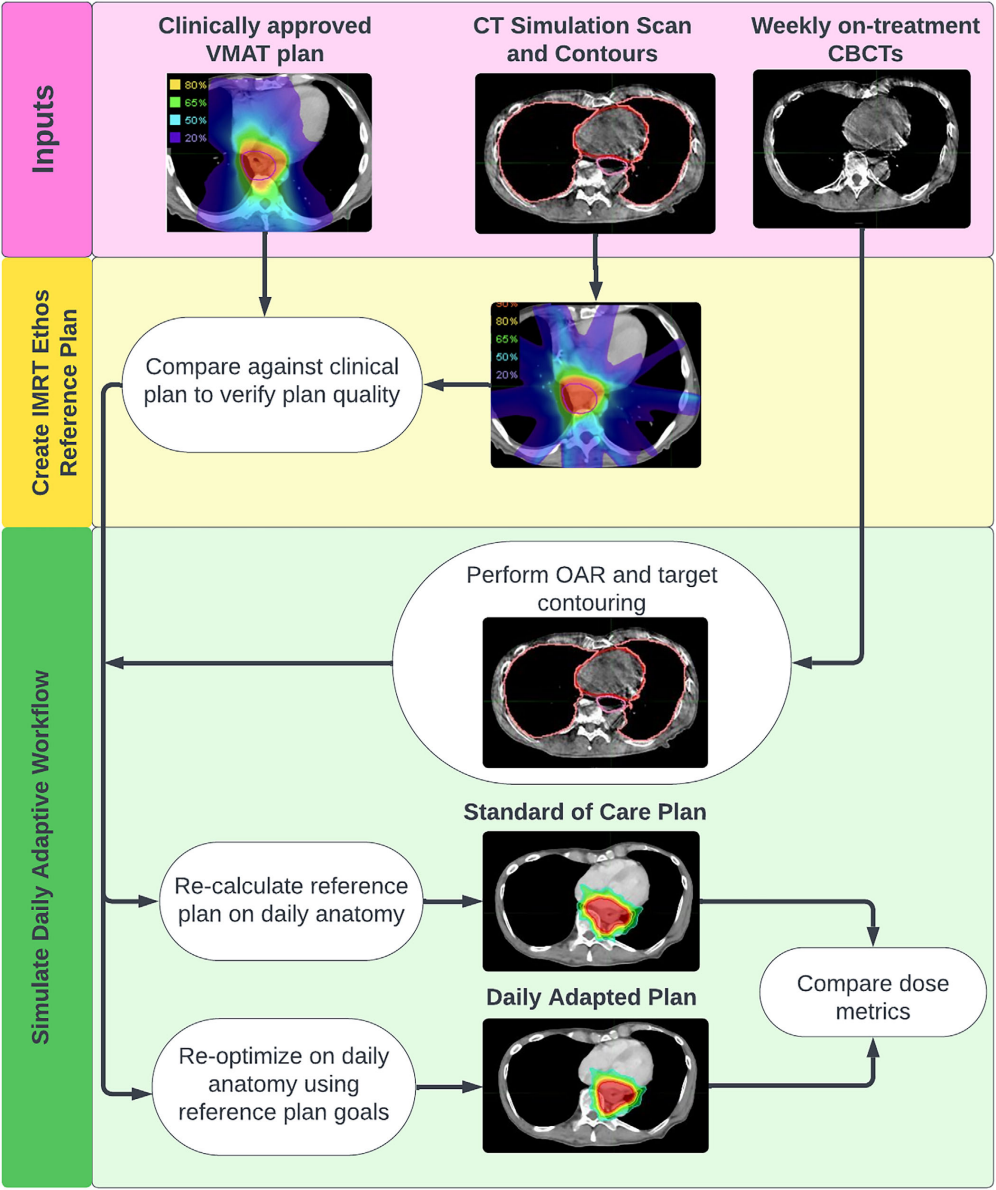
Workflow to simulate standard of care and adaptive doses calculated in this study. For each patient, the above procedure is repeated using five different retrospective CBCTs taken at weekly timepoints to assess the impact of adaptive RT over the course of treatment.

Simulated adaptive sessions were performed as shown in Figure 2 below and as previously described in the literature by several groups.^11^, ^12^, ^13^, ^14^, ^15^, ^16^, ^17^ Using the reference plan developed as discussed in the previous section, five adapted sessions were simulated for each of the 19 patients using their weekly CBCT images. The weekly CBCT was input into the Ethos version 2.0 emulator as the “acquired image.” From there, influencer and OAR structure contouring was performed which consisted of the lungs, heart, esophagus, and stomach. The CTVs were manually edited on each axial slice to match the anatomy covered in the patient's originally approved clinical plan. The PTVs were generated from the CTVs by applying a 5 mm margin on the CTV (following institutional guidelines). Then the adapted plan was optimized and compared to the standard of care (SOC) plan (reference plan fluence recalculated on the anatomy of the day). All contours were reviewed by an MD that routinely treats this site. For the SOC daily dose, the dose metrics were recalculated on the daily anatomy thus ensuring consistency in organ volumes for metric comparison. Both the adapted and SOC plan dose were calculated on a synthetic CT automatically generated by the Ethos adaptive workflow by deforming the reference planning CT to the treatment sessions’ CBCT. All session data including both the adapted and SOC doses were exported from the Ethos treatment management system and imported into MIM software (Cleveland, OH) for analysis.

### 2.4 Dosimetric analysis

Metrics analyzed were taken from Liu et al.^5^ For each patient, the metric values were averaged across their five adapted plans and their five SOC plans. Then for each patient, the percent change in metric was calculated using the equation below where reported changes in metrics are the average of the five fractions. Then for each metric, the percent changes were evaluated for significance using a Wilcoxon signed rank test and *p*‐value < 0.01.

(1) $$Percent\;Change\;=\;\frac{AdaptedMetric-SOCMetric}{SOCMetric}*100\%$$

### 2.5 Modeling

From the dosimetric analysis, seven patients were classified as having high benefit. These patients were classified if they had statistically significant reductions in any normal tissue metrics while not having any statistically significant increases in any of these metrics. A nearest neighbor classifier (KNN) was trained with 19‐fold cross validation using the leave‐one‐out method to predict which patients were high benefit from their original CT simulation scan and RT structure set. All modeling was done in MATLAB Version 2024 (Mathworks Inc, Natick, MA) using the Classification Learner module. Features tested as potential predictive covariates included all OAR volumes, PTV Volumes, and PTV‐OAR overlap volumes. Feature selection was performed by selecting all predictors that were statistically significant (*p* < 0.01) using the Kruskal Wallis feature ranking algorithm. Bayesian optimization with 1000 iterations and an expected improvement acquisition function was used to optimize the KNN hyperparameters. These included the number of neighbors (1–6), distance metric (Euclidean, Jaccard, Hamming, Cubic, Spearman, Correlation, Cosine, Mahalanobis, Chebyshev, and City Block), distance weight (Equal, Inverse, or Squared Inverse), and data standardization (yes or no).

## 3 RESULTS

### 3.1 Ethos reference plan generation

**FIGURE 3:**
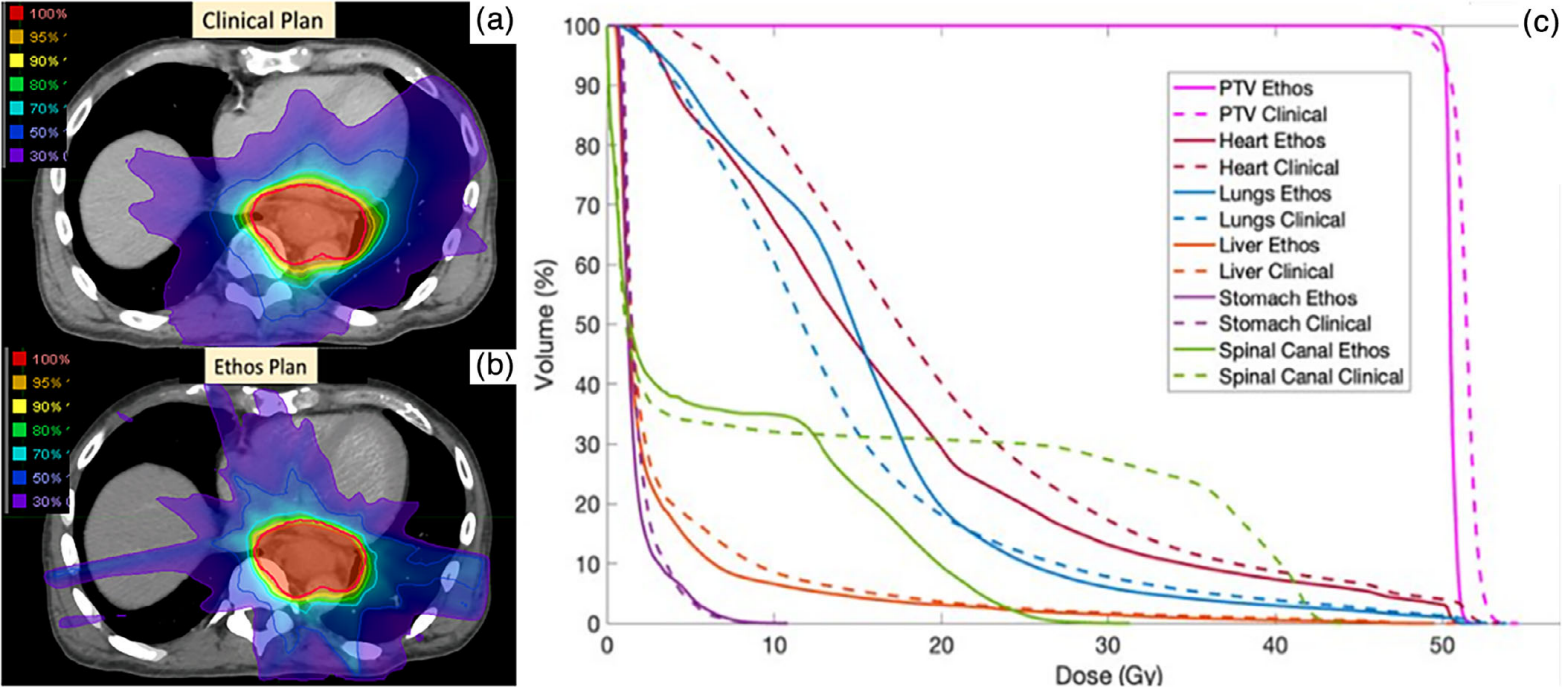
Example of Ethos and clinically‐approved plans’ equivalence for 1 subject. (a) The clinically approved plan using 2‐arc VMAT, (b) Ethos template‐generated plan using 9‐Field IMRT (for optimization speed during adaptive sessions), (c) DVH for both plans showing PTV coverage and relevant OARs. The Ethos template‐generated plan was dosimetrically equivalent or better than the clinical plan that was approved across all relevant metrics).

**FIGURE 4:**
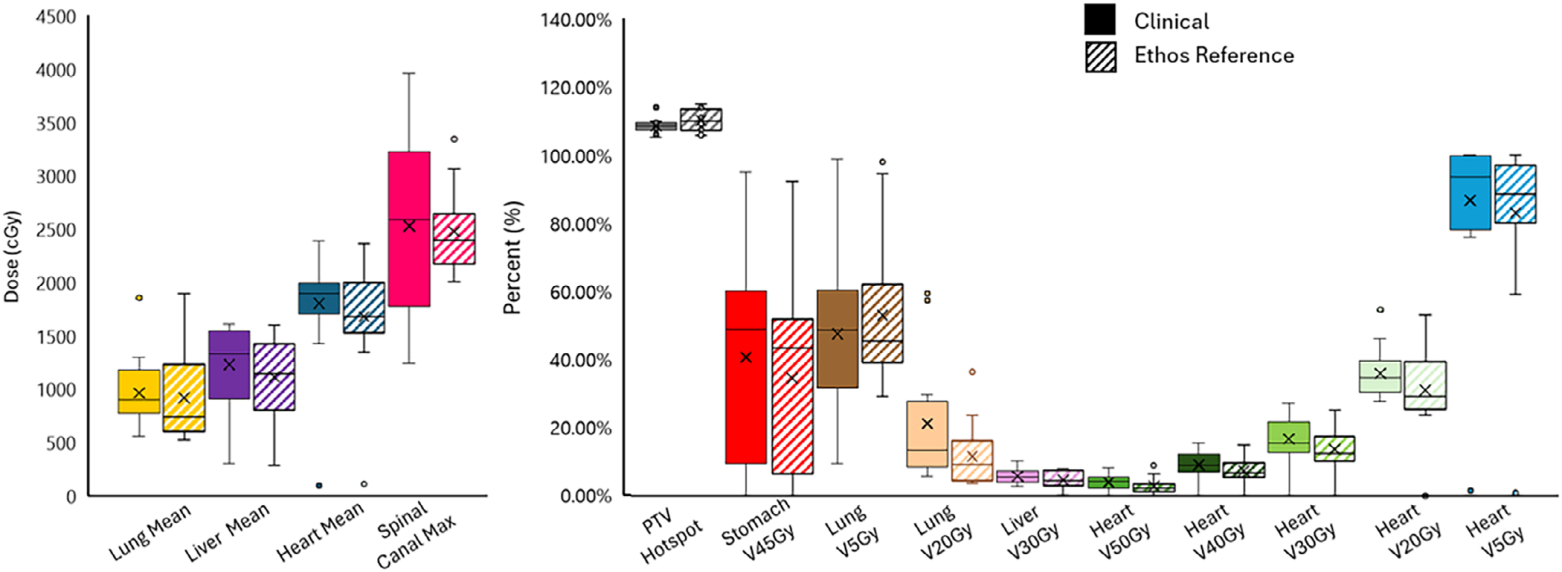
Boxplots showing the metrics analyzed across all patient plans for the clinically approved plans (solid) compared to the Ethos reference plans (striped). The Ethos reference plans performed comparably or better than clinically approved plans across all metrics for all patients.

The IMRT plans generated as the SOC plans in the Ethos TPS were found to be clinically acceptable and of equivalent plan quality to the clinically treated VMAT plans. An example from one subject is shown in Figure 3. While there are differences due to the change in beam geometry, the DVH shows that PTV coverage is maintained while critical organs at risk are still adequately spared. Across all subjects, plan metrics were comparable or better as shown in Figure 4.

### 3.2 Dosimetric changes

**FIGURE 5:**
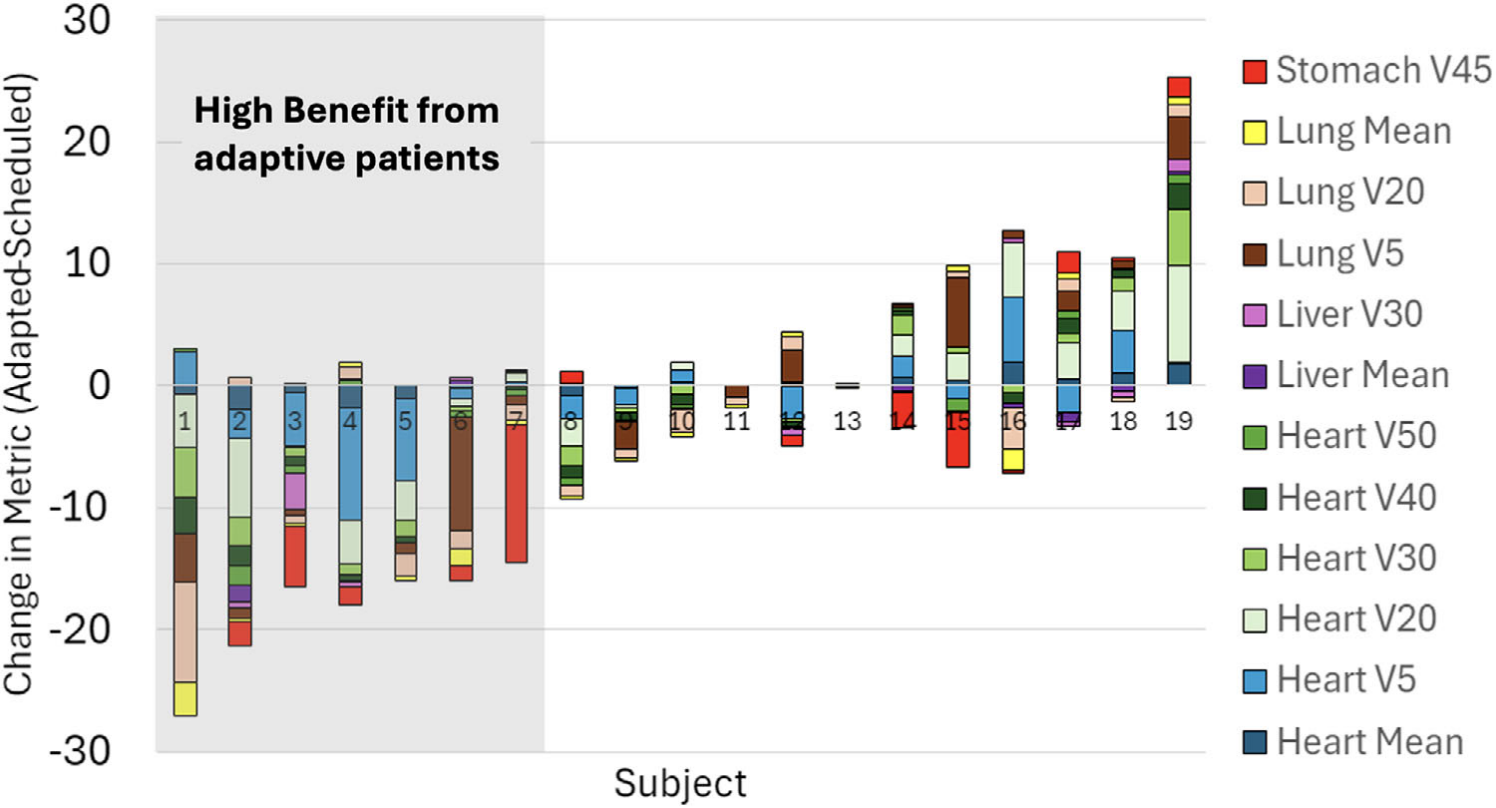
Patient‐specific benefit from daily adaptive. Each stacked bar is a different patient showing the change in each metric analyzed between the daily adaptive and daily SOC plans. Patients are ordered by cumulative benefit (highest benefit to least).

The magnitude of the changes in each metric varied across the 19 patients analyzed. Results for all patients are summarized in Figure 5. Figure 5 shows a holistic representation of the benefits or lack thereof from daily online adaption. The percent change in each metric is color coded and represented in a stacked bar chart to highlight any tradeoffs, if any, that were made in sparing different OARs. Patients with all bars showing negative changes in metrics represent patients who benefited from daily online adaption on every OAR metric and patients with all positive bars represent those who did not. For some patients, there are a combination of positive and negative metrics. For these patients, tradeoffs were made by the optimizer during adaption where the daily adaption caused one organ to be spared more than the SOC plan while another OAR received more dose than their SOC plan. Overall, some patients experienced OAR benefits while others did not.

**FIGURE 6:**
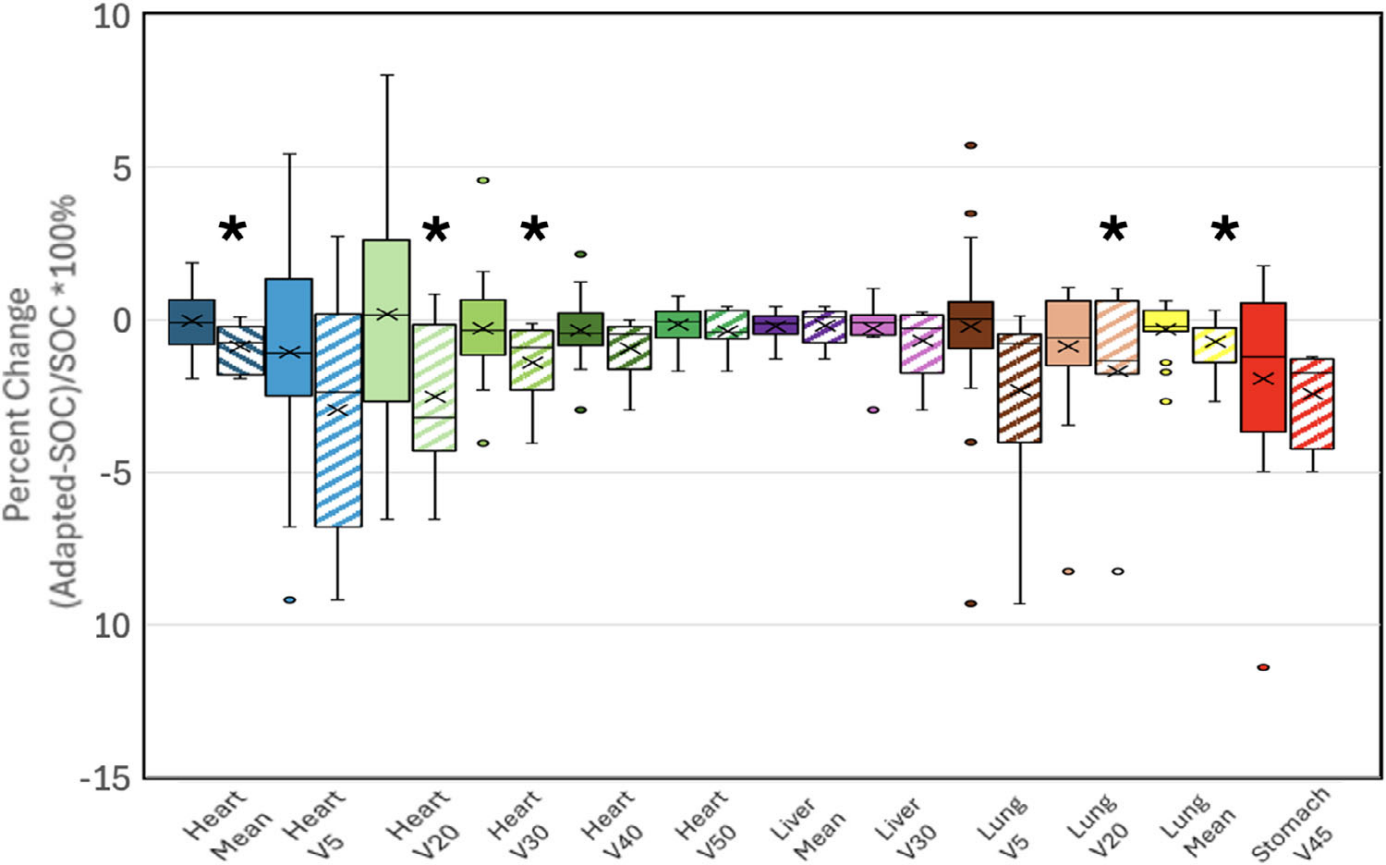
Patient‐Specific benefit from daily adaptive versus daily SOC doses. For each metric, paired boxplots are shown for the entire study population (left/solid) and the high benefit patient cohort (right/striped). Metrics that were statistically significant in the adapted plans versus SOC (*p* < 0.05) are denoted by *.

Patients labelled 1–7 on Figure 5 were classified as having high benefit from adaptive RT. Figure 6 shows a box plot of these patients’ metrics compared to the entire patient cohort‘s metrics. There were five metrics where the high‐benefit patient cohort saw statistically significant improvements in a metric from adaptive RT compared to SOC (heart mean, heart V20, heart V30, lung V20, and lung mean).

### 3.3 Modeling

The results of predictive co‐variate analysis are shown in Table 1. Only the Stomach‐PTV50 overlap and Liver‐PTV50 overlap were found to be statistically significant predictors. The KNN model was trained with optimal hyperparameters displayed in Table 2. These hyperparameters and the two co‐variates resulted in a model that performed with 89.5% accuracy when evaluated with LOOCV as shown in Table 3.

**TABLE 1:** Feature selection results. Highlighted features were used in model as predictors. OL = PTV overlap.

Feature	*p*‐Value
Stomach‐PTV50 OL	0.006
Liver‐PTV50 OL	0.010
Heart‐PTV50 OL	0.021
Heart volume	0.030
Stomach volume	0.030
Stomach‐PTV45 OL	0.038
Lung‐PTV50 OL	0.043
PTV50 volume	0.187
PTV45 volume	0.339
Liver‐PTV45 OL	0.515
Heart‐PTV45 OL	0.668
Lung‐PTV45 OL	0.077
Liver volume	1.000
Lung volume	1.000

**TABLE 2:** Optimal hyperparameters.

Parameter	Value
Number of neighbors	2
Distance metric	City block
Distance weight	Equal
Data standardization	No

**TABLE 3:** Optimal model performance.

Metric	Value
Accuracy	89.5%
TPR	100%
FPR	0%
FNR	15.4%
TNR	84.6%

### 3.4 Timing data

**FIGURE 7:**
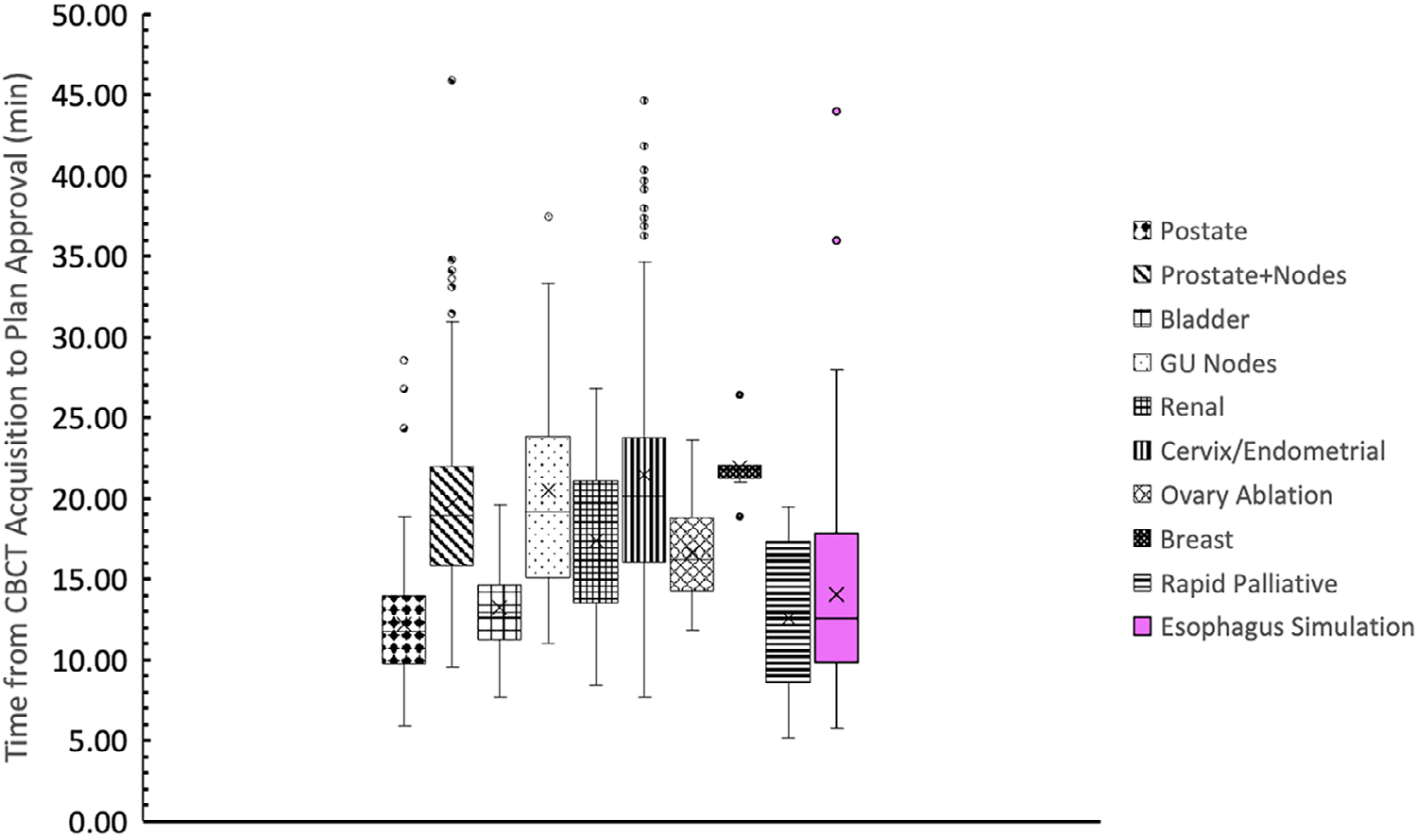
Timing date for routinely adapted sites at our institution as well as from this simulated adaptive study.

The time elapsed from CBCT acquisition to plan approval during the simulated adaptive fractions for the esophagus patient is presented in Figure 7 and compared to the time required for disease sites routinely prospectively adapted at our institution. This time represents the time taken to perform contouring, target review, plan calculation, and plan review. These are the steps that are additions to the standard of care IGRT workflow and represent the time burden incurred by ART. Esophageal ART was on average 14.1 min which is shorter than the average time required at our clinic for many disease sites such as cervix (21.9 min) or prostate and nodes (19.7 min).

## 4 DISCUSSION

### 4.1 IMRT plan quality

Overall, clinically acceptable plans were able to be created using IMRT and the Ethos TPS instead of our clinical standard VMAT and Eclipse optimization. The results in Figure 4 show that for each metric analyzed, the distribution of the metrics across subjects was not statistically different from the original plans. In looking at individual plans, we observed in some case the Ethos reference plans were even of higher plan quality (had lower OAR doses without sacrificing PTV coverage) than the clinical plans. Inherently, IMRT plans and VMAT plans will have some differences such as the IMRT plans having less low dose wash over the body. This was observed in our comparisons with many organs DVH's showing less low dose but similar or slightly higher volumes of higher dose in organs where beams entered through. However, all clinical metrics were met in all plans without sacrificing PTV coverage which was consistently normalized to 95 % of the volume recieving the Rx dose. In our subsequent comparisons, the difference between this reference plan and the adapted plan was used to calculate benefit rather than the clinical plan versus adapted plan difference.

### 4.2 Dosimetric benefits

The results in Figure 5 indicate large variability in each subject's benefit from adaption. Subjects 1–7 experience reductions in multiple metrics without an equivalent increase in other metrics making them classify as subjects who received high benefit from adaption. Some of the reductions were large in magnitude such as an over 6 % reduction in V20 for the lung or a 1.7 Gy reduction in the mean heart dose. These reductions have been shown by previous work to correlate to reduced probability of toxicities. Darby et al reported the relative risk for ischemic heart disease is 7.4% per 1 Gy mean heart dose^18^ which would mean the reductions observed in this study could mean as high as a 12.6% reduction in relative risk of ischemic heart disease. Furthermore, lung V20 has been shown in many studies to relate to risk of radiation pneumonitis.^19^ Additionally, as seen in Figure 6, when analyzing this subgroup of subjects separately, statistically significant reductions in the heart mean, heart V20 and V30, lung V20, and lung mean were seen when comparing adaptive to SOC plans.

However, it is clear not all patients benefit equally. For some subjects, benefits to individual metrics are small, or a benefit to one organ is paired with increased dose to another critical metric. Analysis of CTV volume change was performed. Although all patients that saw large reductions in volume (some up to a 50 percent reduction) benefited, no clear benefit versus nonbenefit correlations were found from those who did not experience large CTV changes. Some patients with little to no volume change in their CTV benefited, while others did not. It therefore appears the adaptive benefit is driven by a combination of organ at risk changes as well as target volume changes. Figure 5 also shows some subjects that appear to be better off treated with the SOC nonadapted plans. This is unexpected, as we would expect the dosimetry metrics of the adapted plan to always be as good or better than the scheduled plan. We suspect this result is due to the goals of optimization set in the Ethos reference plan. From our experience with our adaptive program thus far, we have found that if the goals are not robust to significant changes in anatomy, the optimizer can be presented with goals that are impossible which causes it to ignore the goal entirely. Additionally, if metrics are meeting hard constraints, the optimizer is not heavily penalized for small dose detriments to OARs especially if it is trying to achieve higher PTV coverage. Further work in refining our planning directive templates would be needed to fully understand this behavior and determine what the true benefit, if any, these patients would receive. Unfortunately, the original planning templates were lost by the manufacturer for this study and thus a new study would need to be performed to assess this. It is also worth acknowledging that different locations of disease may result in different outcomes. For example: lower esophagus patients may face different OAR sparing concerns than upper esophagus patients. Because of the distribution of disease locations in our subject pool (wide majority lower esophagus), we were unable to assess this or stratify analysis. However, future investigations with access to larger study cohorts should consider this.

### 4.3 Clinical feasibility

From a clinical feasibility view, one of the primary considerations is the additional time for adaption compared to the standard IGRT workflow. This is important from both a clinic scheduling perspective but also an uncertainty perspective. The longer the patient is required to lay on the table increases the risk of patient motion or internal changes to the anatomy. When adapting, it is important to assure that the anatomy contoured on is in fact the anatomy that will be treated on. The results shown in Figure 7 suggest that the time required to adapt is reasonable and comparable to other sites that are routinely adapted at our institution. It is important to note however that the times reported for this study were from simulated adapted data. Therefore, these times do not take into account any added time from things like waiting on a physician to come review contours, needing to re‐image or recontour because a patient moved, etc. whereas the other sites times in Figure 7 are reported from clincial data that does include these delays. However, from our experience, the largest factors in the time it takes to adapt come from features of the plan such as volume of the targets and number of OARs. For esophageal patients, the OARs (heart, lungs, stomach, liver, and spinal canal) are relatively simple to contour compared to a structure like the sigmoid or bowel loops. Additionally, the overall volumes of targets are small taking less time to draw and review. Therefore, despite our reported times not fully encompassing clinical reality, we do feel it suggests the time to adapt these patients is reasonable and comparable to other sites we routinely adapt.

However, another consideration is resource allocation. Although there may be some benefit to many IMRT/VMAT patients, in a busy clinic, it is impractical to treat every patient adaptively. However, the alternative of needing to offline adapt patients that see large anatomic changes is also straining on clinical resources. Adaptive treatments take additional time on the machines and require extra staffing. Many centers take the approach of holding a set number of adaptive slots each day which corresponds to the clinic's capacity for providing these additional personnel and the additional machine time. Therefore, it is imperative that the patients receiving the adaptive treatment slots are those who need and will benefit from it the most. From the results shown in the modeling section, it appears there may be a way to prospectively select these patients for this site, however it is important to note that this model was internally validated. For the most confident predictions to optimize resource allocation, the model will need to undergo additional validation before being used to recommend esophageal cancer patients for adaptive versus standard of care IGRT. Furthermore, additional studies correlating the improvements in dosimetric parameters with actual changes in grades of adverse events experienced by patients would help to validate these models and the clinical benefit of adaptive radiation.

### 4.4 Study limitations

Although this study shows the potential for adaptive benefit in patients with esophageal cancer, we recognize that there are limitations to the approach. One is that this study did not assess the possibility of any intrafraction variation. This is a unique additional uncertainty on top of the normal setup uncertainties seen in all IGRT that is increased during adaptive workflows due to the increased time the patient spends on the couch. This was not thoroughly analyzed due to the nature of the study and data available for these patients. It is well‐known that changes in anatomy during the process of adapting and treating could result in clinically significant differences in doses delivered to targets and OARs. Many centers, including ours, use confirmation imaging performed at the end of plan calculation to ensure the variation from the CBCT contoured and planned on is not different from the CBCT of the anatomy being treated. Since these patients were not actually treated adaptively, we do not have these additional images to analyze. We recommend methods similar to this be used if implementing any adaptive treatment clinically.

Another limitation is in the sample size of the study prevents any wide‐scale conclusions. The purpose of this study was to be a pilot study only. Further studies with larger cohorts are needed to fully understand the nuances of each patient's clinical situation and whether adaptive RT is appropriate for them. Specifically, subgroup analysis would be helpful to determine which groups of esophageal cancer patients are likely to benefit. Furthermore, our sample size limits the generalization of the model. Our model was trained with cross validation techniques to avoid overfitting, however it is still possible some overfitting is present. A validations study should be performed to confirm the performance.

## 5 CONCLUSIONS

This work shows the clinical feasibility and potential benefit to esophageal patients from daily ART. Esophageal patients’ tumors and surrounding anatomy can change drastically over the course of treatment (Figure 1) and can require offline adaption. This unpredictable and urgent need for offline replanning can be disruptive to clinical workflows compared to scheduled, fast, daily adaptation. Daily adaptation is also resource‐intensive and as seen in our study not all patients have significant benefit. However, in this work, we developed a predictive model capable of identifying these potentially high‐benefit patients based on pretreatment characteristics for efficient resource allocation. Adaptive planning is likely not required for all esophageal cancer patients, but may be highly beneficial for a subset. More studies with larger patient cohorts are needed to fully understand this nuance.

## AUTHOR CONTRIBUTIONS

The authors confirm contribution to the paper as follows: *Study conception and design*: Antonia Kubiatowicz, Michael Sherer, Ayaka Owaga, and Xenia Ray. *Data collection*: Antonia Kubiatowicz. *Analysis and interpretation of results*: Antonia Kubiatowicz, Michael Sherer, Ayaka Owaga, and Xenia Ray. *Draft manuscript preparation*: Antonia Kubiatowicz and Xenia Ray. *Manuscript review*: All authors.

## CONFLICT OF INTEREST STATEMENT

Xenia Ray received honoraria, has a lab services agreement with, and is the Physics PI on a clinical trial sponsored by Varian, a Siemens Healthineers Company. Andrew Sharabi reports being a consultant/advisory board member for AstraZeneca, Primmune, Merck, and Jounce Therapeutics; reports receiving commercial research grants from Varian Medical Systems/Siemens Healthineers and Pfizer. A.B. Sharabi holds ownership interest in Toragen Inc. and Advanced B‐cell Therapeutics outside of submitted work.

## Supporting information

Supporting Information
