# Supplementary material for: Assessing dosimetric benefit from daily online adaptive radiation therapy for esophageal cancer
Source: J Appl Clin Med Phys. 2025 Sep 23;26(10):e70244. doi: 10.1002/acm2.70244 (PMC12457208; doi:10.1002/acm2.70244)
Supplement: Supplementary file 1 — Supporting Information [file ACM2-26-e70244-s001.pdf]

Structure Plan	Type	Constraint	Goal
SpinalCanal	OAR	D0.03cc ≤ (Up to 47 Gy is allowed, but below 45 Gy preferred)	4000-4500cGy
SpinalCanal	OAR	Max (Tracking only)	cGy
SpinalCanal_PRV5	OAR	D0.03cc ≤ ~ (Soft)	5000cGy
BowelSpace	OAR	D0.03cc ≤	5400-6000cGy
BowelSpace	OAR	V4500cGy ≤ ~ (Soft)	200cc
Esophagus	OAR	D0.03cc ≤	6000cGy
Heart	OAR	Mean ≤	2000-3500cGy
Kidneys	OAR	Mean ≤ ~ (Soft)	1400cGy
Liver	OAR	Mean ≤ (Non-Cirrhotic)	3000cGy
Liver	OAR	Mean ≤ (Cirrhotic)	2800cGy
Stomach	OAR	D0.03cc ≤	6000cGy
Lungs	OAR	V2000cGy ≤ ~ (Soft)	30%
Lungs	OAR	V500cGy ≤ ~ (Soft)	60%
Lungs	OAR	Mean ≤ ~ (Soft)	2000cGy
PTV50	Target	V100% ≥ ~ (Soft)	95%
PTV50	Target	Max ≤ ~ (Soft)	107-115%
